# Assessment of adversity quotient®: decoding strength and struggle among faculty members of a medical university: a cross-sectional study

**DOI:** 10.1097/MS9.0000000000005308

**Published:** 2026-07-15

**Authors:** Preh Abro, Muhammad Ilyas Siddiqui, Fnu Bhawna, Zunaira Solangi, Sadam Hussain, Sidhant Ochani, Kaleem Ullah, Salah Termos, MD Asaduzzaman Nur

**Affiliations:** a Department of Community Medicine, Bibi Aseefa Dental College, SMBBU Larkana, Larkana, Sindh, Pakistan; b Faculty of Community Medicine & Public Health Sciences, Liaquat University of Medical & Health Sciences, Jamshoro, Sindh, Pakistan; c College of Public Health, Ziauddin University, Karachi, Pakistan; d Department of Hepatobiliary Surgery, Al-Amiri Hospital, Kuwait City, Kuwait; e Department of Hepatobiliary Surgery, Enam Medical College and Hospital, Dhaka, Bangladesh

**Keywords:** Adversity Quotient®, demographic factors, medical university faculty, strength and struggle

## Abstract

**Objective:**

To assess the Adversity Quotient® (AQ®) among medical teaching faculty members by evaluating overall AQ® and its dimensions – Control, Ownership, Reach, and Endurance – while analyzing the influence of demographic variables and examining interrelationships among the CORE components.

**Material and methods:**

A descriptive, cross-sectional survey was carried out at Shaheed Mohtarma Benazir Bhutto Medical University (SMBBMU), Larkana, Pakistan, between August 2024 and January 2025. A stratified random sample of 157 full-time faculty members, proportionately drawn from six constituent colleges and institutes, was recruited, with eligibility requiring at least 2 years of service. AQ® was measured using the validated Stoltz Adversity Response Profile, and demographic information was obtained via a standardized proforma. All data were entered into IBM SPSS Statistics v30.0 and summarized with descriptive statistics (means, standard deviations, and frequencies). Spearman’s rank-order correlation was used to explore associations between AQ® scores (overall and CORE subscales) and demographic variables, with two-tailed significance set at *P* < 0.05.

**Results:**

The mean total AQ® among faculty was 138.4 ± 20.4, indicating an overall average resilience level. Most respondents scored average in Control (36.3%), Reach (38.9%), and Endurance (46.5%), but a significant proportion (42.7%) scored low in Ownership, highlighting a potential area for development. No significant correlations were found between AQ® and age, gender, education level, or academic rank. However, marital status and work setting showed weak but significant associations with certain CORE dimensions, particularly Control and Ownership. Length of service was positively correlated with total AQ®. Notably, strong internal consistency was observed between paired dimensions – Control with Ownership (*ρ* = 0.484) and Reach with Endurance (*ρ* = 0.289), both statistically significant (*P* < 0.001).

**Conclusion:**

This assessment of AQ® offers meaningful insight into faculty members’ adaptive capacity when facing challenges. The notably low scores in Control and Ownership highlight key areas for targeted development. Enhancing these dimensions through structured interventions may significantly strengthen overall resilience, empowering faculty to navigate adversity more effectively in both professional and personal domains.

**Graphic abstract:**

Graphic abstract available at: http://links.lww.com/MS9/B279

**Figure:**
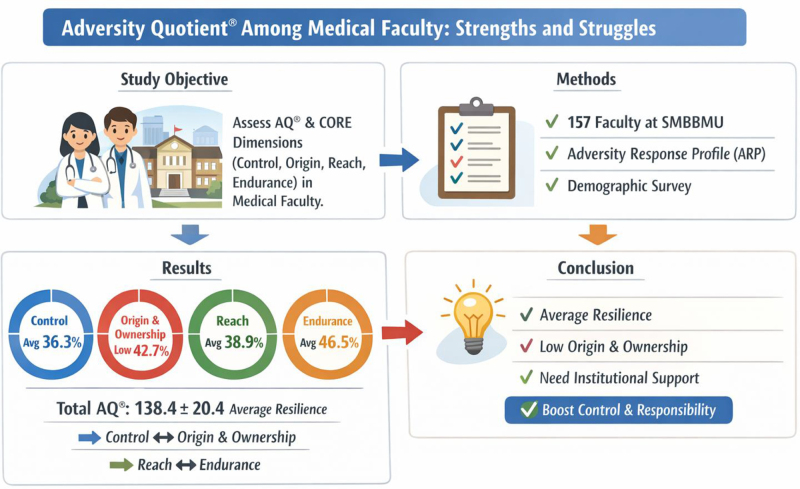

## Introduction

In the ever-evolving field of medical education, faculties face numerous challenges arising from their teaching duties, clinical work, research expectations, and administrative tasks. Juggling these diverse responsibilities frequently leads to increased stress, emotional exhaustion, and difficulty maintaining a healthy work-life balance, all of which negatively affect their overall well-being and effectiveness as professionals^[^1,2^]^. Although conventional measures like Intelligence Quotient (IQ) and Emotional Intelligence (EI) have been extensively researched in education and work settings, increasing attention is being given to Adversity Quotient® (AQ®). Developed by Paul Stoltz, AQ® evaluates a person’s ability to endure, manage, and bounce back from challenges^[^3,4^]^. The AQ®, developed by Stoltz, provides a theoretical framework for understanding how individuals respond to challenges, setbacks, and stress. AQ® encompasses four core dimensions: Control, Origin & Ownership, Reach, and Endurance. Control reflects the perceived ability to influence or manage adverse situations. Origin & Ownership captures the extent to which individuals attribute responsibility for adversity and take charge of responding to it. Reach refers to how far the impact of adversity is allowed to spread into other areas of one’s life or work, while Endurance assesses whether individuals perceive adversity as temporary or long-lasting[5].

HIGHLIGHTS

- The overall Adversity Quotient® (AQ®) among medical faculty was average (mean score: 138.4 ± 20.4), with most respondents scoring within the mid-range across CORE dimensions.
- No significant associations were found between AQ® and age, gender, education, or academic rank. However, marital status and work setting had weak but significant associations with Control and Ownership.
- A considerable number of faculty scored low in the Control (25.5%) and Ownership (42.7%) dimensions, highlighting key areas needing targeted resilience-building interventions.
- Strong correlations were observed between Control and Ownership (*ρ* = 0.484, *P* < 0.001) and between Reach and Endurance (*ρ* = 0.289, *P* < 0.001), indicating interdependence within AQ® subcomponents.

Studies indicate that those with elevated AQ® levels demonstrate enhanced resilience, improved adaptability, and stronger motivation when facing workplace difficulties[6]. Within academic and healthcare environments, higher AQ® levels correlate with more effective stress-coping mechanisms, superior instructional outcomes, and increased professional fulfillment^[^7,8^]^. Although AQ® has garnered international attention, it remains a relatively neglected research area in South Asia, with particularly scarce investigation within Pakistan’s medical education framework. Existing regional studies from neighboring nations like India and Indonesia have predominantly examined AQ® in student cohorts or broad occupational groups, with minimal focus on educator or healthcare professional populations^[^9,10^]^

In Pakistan, there is a notable lack of empirical research examining AQ® among medical faculty, despite their critical role in training future healthcare practitioners. Although existing studies in the region highlight faculty burnout, job dissatisfaction, and coping mechanisms, they fail to systematically assess resilience using validated psychometric measures such as AQ®^[^11,12^]^. Moreover, the relationship between demographic factors, including age, gender, marital status, and professional tenure, and AQ® (along its CORE dimensions) remains poorly understood. This knowledge gap impedes the design of evidence-based interventions to enhance faculty resilience and institutional support frameworks**^[^**4,8^]^. This study aims to fill this critical gap by assessing the AQ® among faculty members of Shaheed Mohtarma Benazir Bhutto Medical University (SMBBMU), exploring their scores across the four CORE dimensions, and analyzing the relationship between AQ® and demographic factors. By decoding both the strengths and struggles embedded in faculty responses to adversity, this research provides evidence to guide professional development programs and support frameworks aimed at enhancing institutional resilience in academic health.

Building upon the identified gaps in the existing literature, the present study was undertaken with clearly defined objectives and hypotheses. The primary objective was to evaluate the AQ® levels among faculty members employed across diverse departments within the university. The secondary objectives were to analyze the association between demographic characteristics and AQ® scores, and to investigate the potential interrelationships among the CORE dimensions of the AQ® framework. This investigation was guided by the hypothesis that demographic variables, specifically age, gender, academic rank, and departmental affiliation, are significantly associated with variations in AQ® levels among faculty members, and that the CORE dimensions demonstrate meaningful interrelationships. By addressing these aims, the study seeks to contribute to a deeper understanding of resilience and adaptability within academic medical settings, thereby informing strategies for faculty development and institutional support. This cross-sectional study has been reported in accordance with the STROBE guidelines[13].

## Materials and methods

This descriptive cross-sectional study was conducted from August 2024 to January 2025 at the Institute of Health Management and Research Sciences, Liaquat University of Medical and Health Sciences (LUMHS), Jamshoro. The study population comprised full-time faculty members from the constituent colleges of SMBBMU, located in Larkana and Sukkur, Sindh. A two-stage sampling method was used to ensure broad representation. In the first stage, all six constituent colleges and institutes were selected through convenience sampling. In the second stage, stratified probability sampling was employed to recruit faculty members from each college proportional to its size. According to SMBBMU’s Human Resources Department, the total faculty strength was 382, distributed across Chandka Medical College (N₁ = 165), Ghulam Mohammad Mahar Medical College (N₂ = 111), Bibi Aseefa Dental College (N₃ = 65), Institute of Physiotherapy (N₄ = 13), Institute of Physiotherapy and Rehabilitation Sciences (N₅ = 14), and College of Nursing (N_6_ = 14). Prior to recruitment, faculty members were approached in person and through call invitations. Participation was voluntary, and written informed consent was obtained. All responses were collected anonymously, and no identifiable information was recorded to maintain confidentiality.

Eligible participants included full-time faculty with a minimum of 2 years of service at SMBBMU, regardless of academic rank, departmental affiliation, or designation. Those with less than 2 years of service, visiting or foreign faculty, individuals from private or affiliated institutions, and those unwilling to participate were excluded. The study investigated demographic variables such as gender, age, marital status, education level, academic rank, length of service, affiliated college, and work area (clinical or basic sciences). The core study variables included the Total AQ® score and its four CORE components: Control, Origin & Ownership, Reach, and Endurance.

Data collection was carried out through structured face-to-face interviews using two validated instruments. The first was a demographic questionnaire capturing relevant background information, and the second was the AQ® Profile (AQP), developed by Paul Stoltz and licensed by PEAK Learning[14]. The AQP includes 20 hypothetical adversity scenarios, with five items per CORE dimension rated on a 5-point Likert scale. Total AQ® scores were calculated by doubling the sum of item scores, with a maximum score of 200. Each interview session lasted approximately 15–20 minutes. All completed questionnaires were reviewed for completeness and accuracy prior to data entry.

### Classification of AQ® performance levels

**Table:** 

Performance level	Control	Origin & Ownership	Reach	Endurance	Total AQ® Score
High	48–50	50	43–50	44–50	176–200
Above average	43–47	47–49	38–42	39–43	158–175
Average	36–42	44–46	30–37	32–38	136–157
Below average	30–35	31–40	25–29	26–31	119–135
Low	10–29	10–30	10–24	10–25	40–118

Classification thresholds were adopted from the Stoltz Adversity Response Profile (ARP) scoring manual. Total AQ® scores were calculated by summing the CORE dimension scores and applying the standardized doubling procedure.

Data were analyzed using IBM SPSS Statistics Version 30.0. Descriptive statistics, including frequencies, percentages, means, and standard deviations, were used to summarize the demographic characteristics and AQ® scores. Spearman’s rank-order correlation was applied to examine relationships between demographic variables and both the overall AQ® and its CORE dimensions, with statistical significance set at *P* < 0.05.

## Results

**Figure 1.**
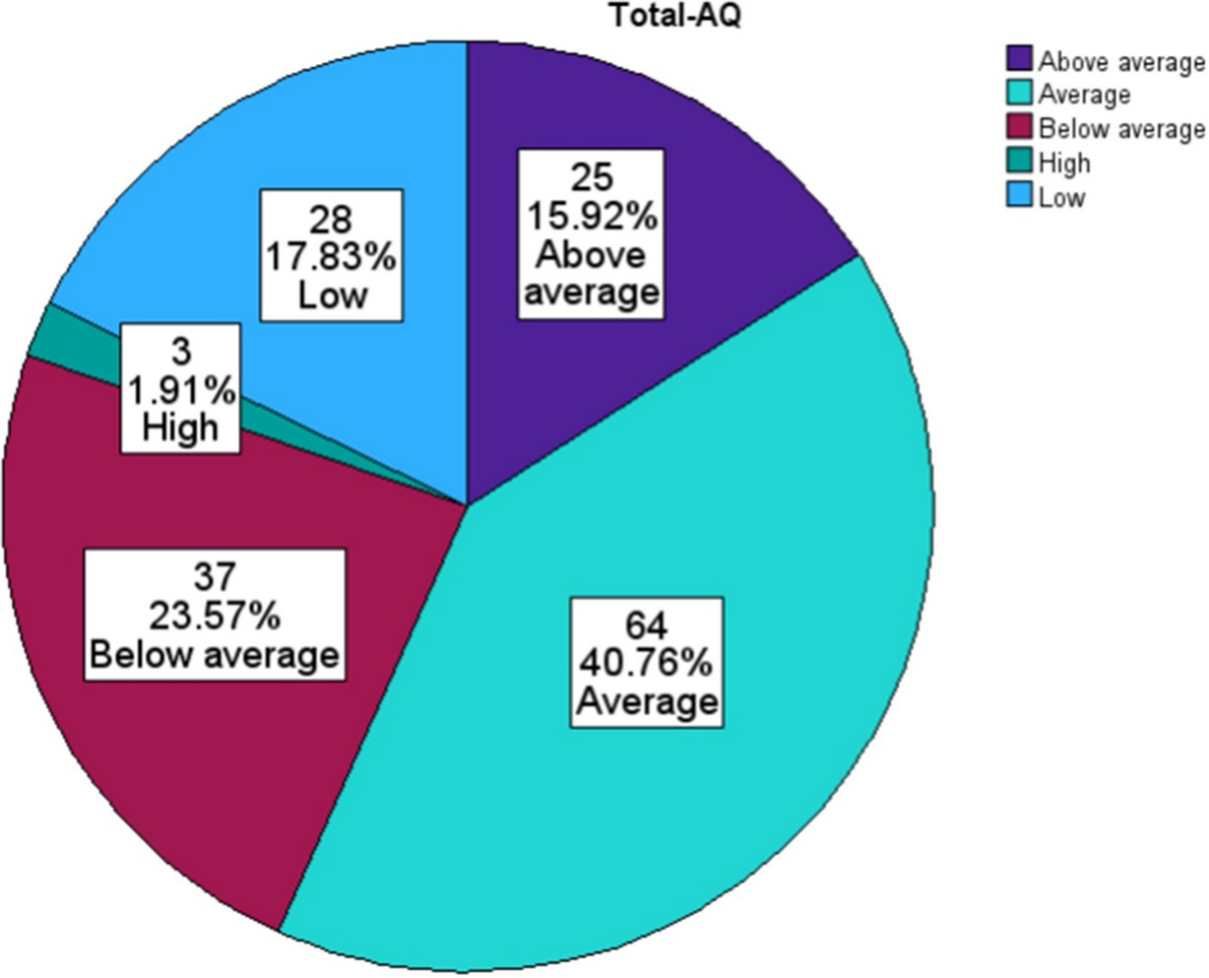
Frequency distribution of the respondents according to the Adversity Quotient®.

A total of 157 faculty members participated in the study. Overall AQ® scores ranged from 64 to 190, with a mean of 138.4 (SD = 20.4), placing most respondents in the “average” category (Table 1). Specifically, 40.8% scored average, 23.6% below average, 17.8% low, 15.9% above average, and only 1.9% high (Fig. 1). These findings suggest that, while resilience was generally moderate, a notable proportion struggled at the lower end of the spectrum.

**Table 1:** Descriptive Statistics for Adversity Quotient® (*n* = 157).

Descriptive Statistics					
	*N*	Minimum	Maximum	Mean	SD
Total-AQ	157	64	190	138.42	20.436
AQ-Control	157	10	48	33.24	7.937
AQ-Origin & Ownership	157	10	50	31.61	9.189
AQ-Reach	157	14	50	36.76	8.146
AQ-Endurance	157	16	50	36.76	6.811

**Figure 2.**
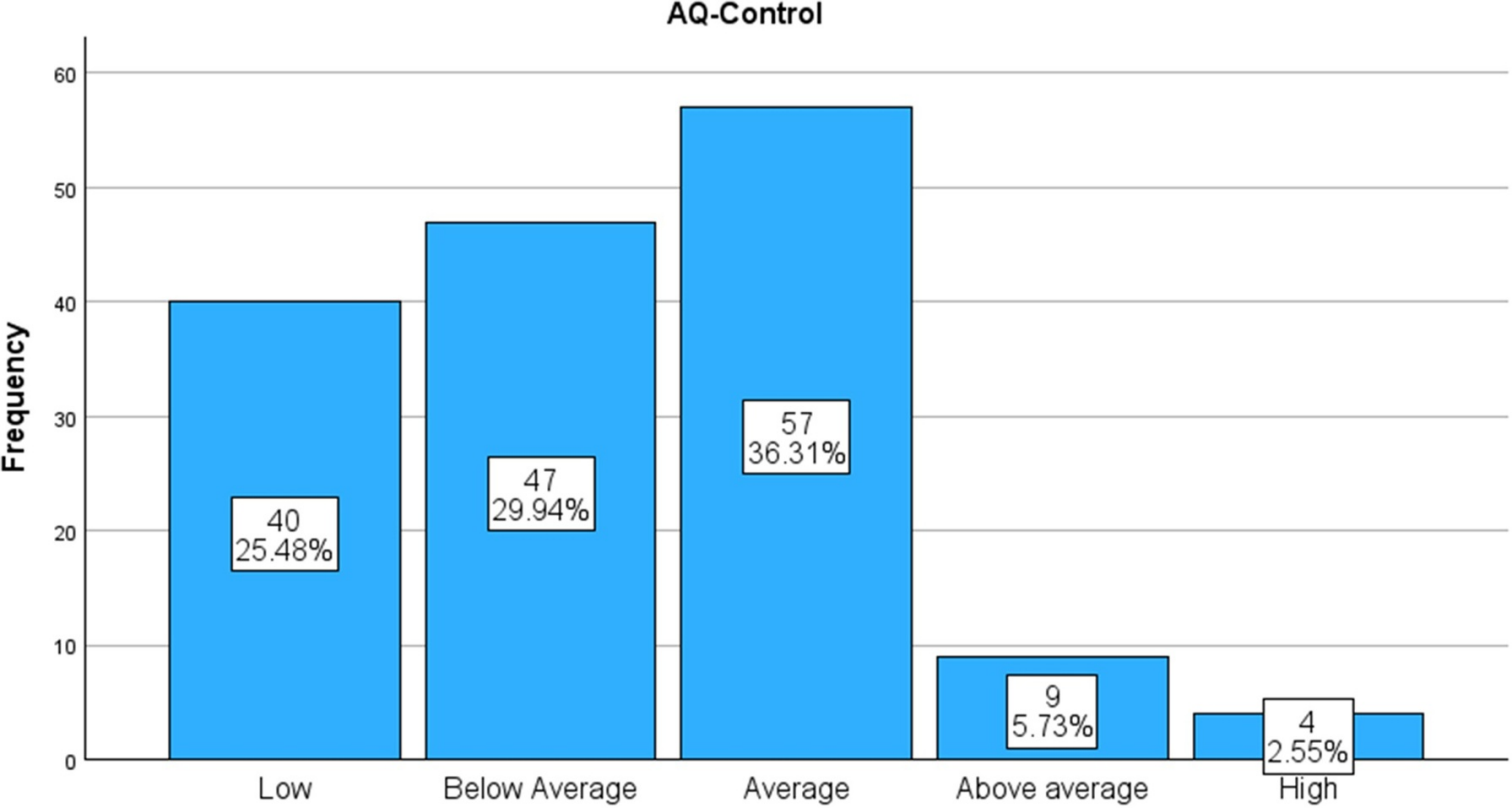
Frequency distribution of the control dimension of Adversity Quotient® (AQ®). The largest group of respondents (36.31%) had average control, while 29.94% scored below average, including 25.48% classified as low. Higher levels of control were less common, with 5.73% above average and 2.55% classified as high.

Across the four CORE dimensions, distinct patterns were observed. In Control, 36.3% scored average, whereas nearly 30% scored below average and 25.5% scored low, indicating limited confidence in managing challenges. Only 8.3% achieved above-average or high scores (Fig. 2; Table 1).

**Figure 3.**
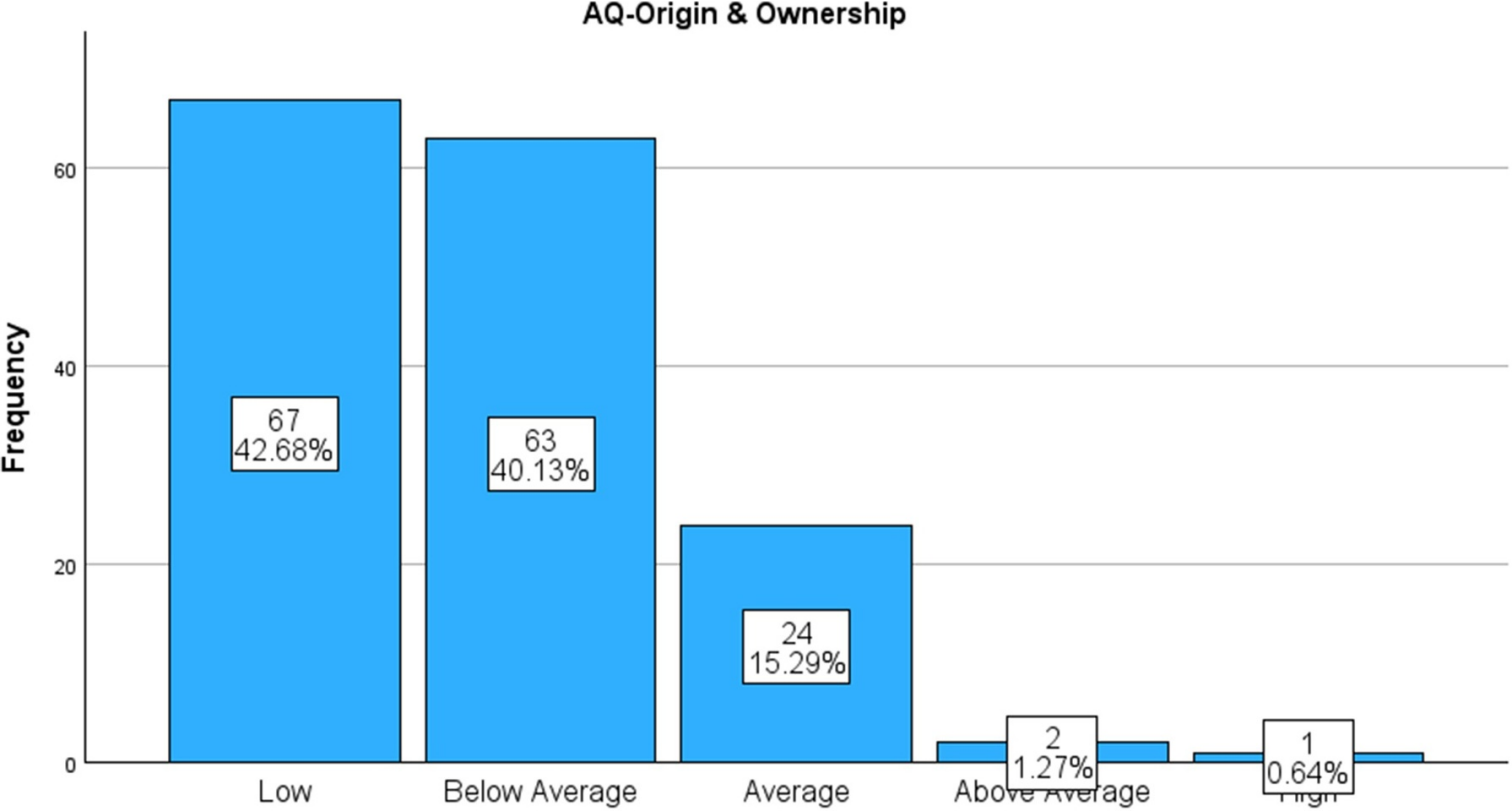
Frequency distribution for the Origin & Ownership dimension. Most respondents scored low (42.68%) or below average (40.13%), while 15.29% were average, 1.27% above average, and 0.64% high.

The Origin & Ownership dimension showed the weakest performance: 42.7% scored low and 40.1% below average, with only 15.3% average and fewer than 2% above average or high (Fig. 3; Table 1). This highlights reduced responsibility-taking and limited perceived influence over adversity.

**Figure 4.**
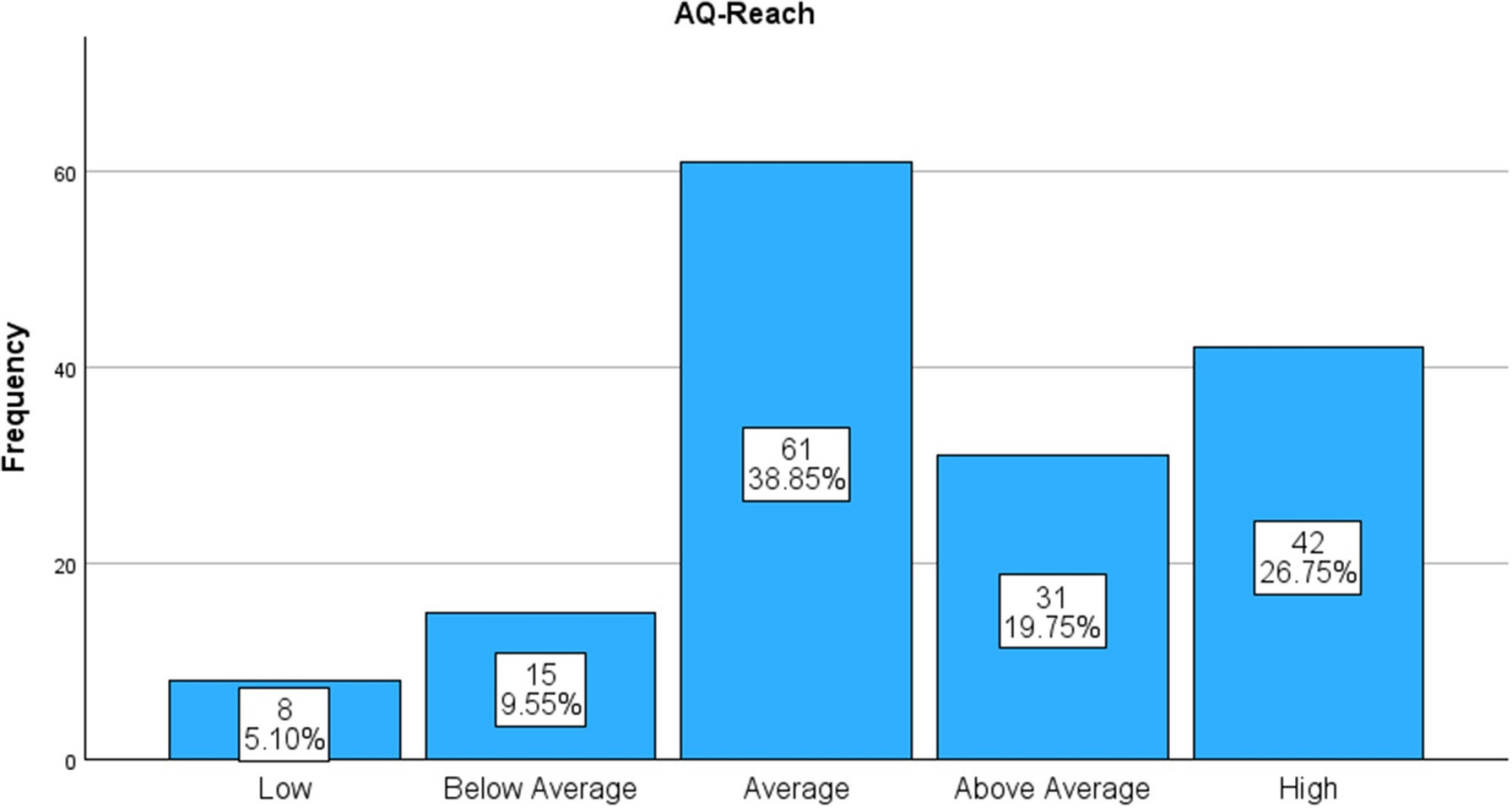
Frequency distribution for the reach dimension. Most respondents scored average (38.85%), followed by high (26.75%), above average (19.75%), below average (9.55%), and low (5.10%).

In contrast, Reach scores were stronger. Over one-third (38.9%) were average, 26.8% high, and 19.8% above average, while only 14.7% fell into the below-average or low categories (Fig. 4; Table 1). This suggests many faculty members were able to contain adversity without allowing it to spread across other aspects of life or work.

**Figure 5.**
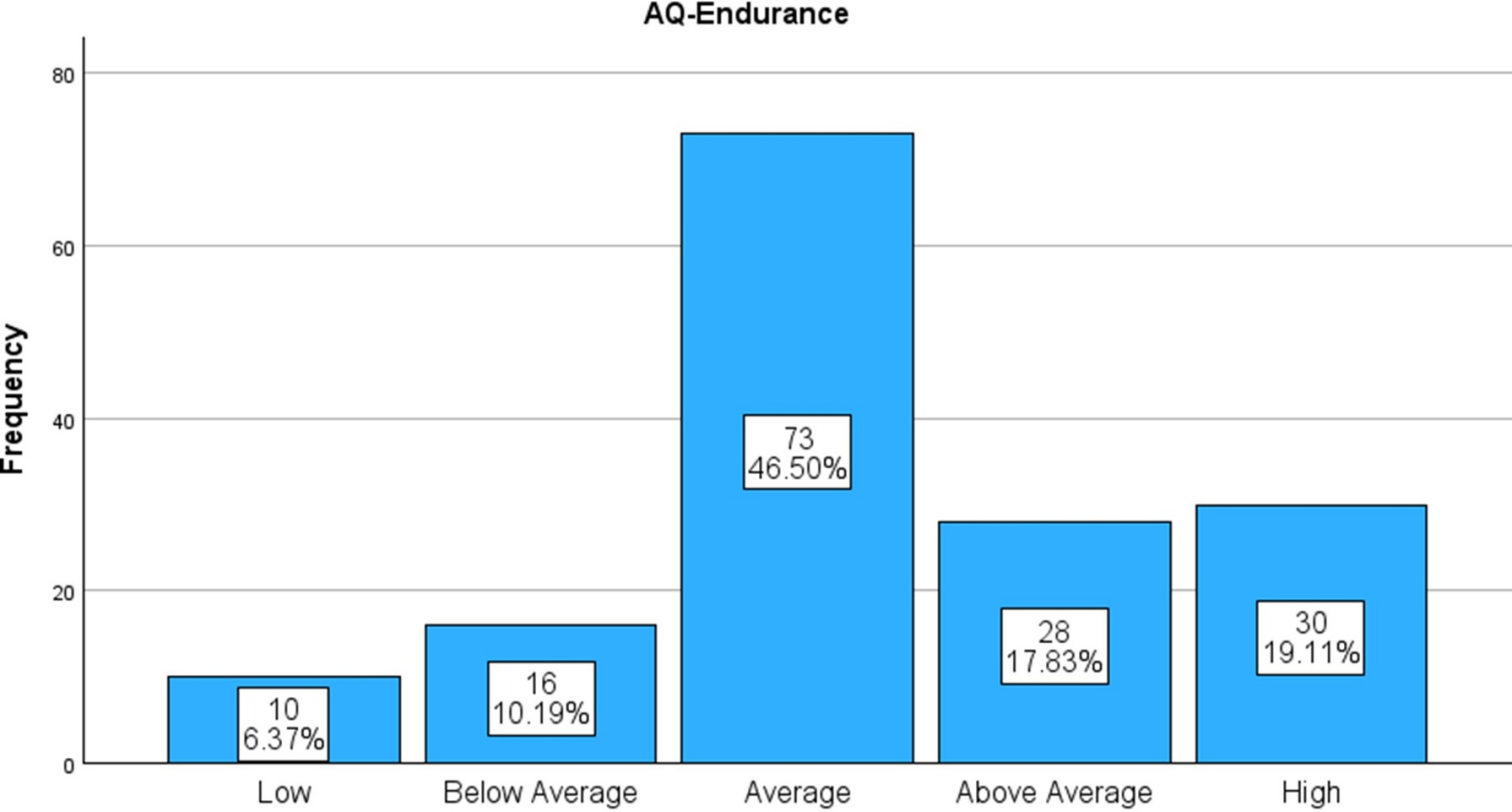
Frequency distribution for the endurance dimension. Most respondents (46.50%) scored average, followed by high (19.11%), above average (17.83%), below average (10.19%), and low (6.37%).

The Endurance dimension reflected moderate resilience, with 46.5% average, 19.1% high, and 17.8% above average. Only 16.6% were below average or low (Fig. 5; Table 1), indicating that most faculty viewed adversity as temporary and manageable.

Correlation analysis (Table 2) revealed no significant associations between AQ® scores and age, gender, education level, or academic rank. However, marital status showed weak but significant negative correlations with Control (*r* = −0.218, *P* = 0.006) and Origin & Ownership (*r* = −0.267, *P* < 0.001), suggesting unmarried faculty reported lower perceived control and responsibility. Similarly, length of service demonstrated a weak positive correlation with total AQ® (*r* = 0.178, *P* = 0.026), indicating resilience increased slightly with tenure. Faculty in clinical departments scored marginally higher in Control compared to those in basic sciences (*r* = 0.176, *P* = 0.028), reflecting the demands of fast-paced decision-making. Exploratory comparisons also showed female faculty had slightly higher median scores in Control and Reach, and senior faculty tended to score higher in Endurance, though these differences were not statistically significant.

**Table 2:** Correlation between Demographic Variables and AQ® and CORE dimensions (*n* = 157).

Variables	AQ®-dimensions	Spearman’s rho coefficient correlation	*P*-Value
Gender	Control	0.006	0.940
	Origin & Ownership	−0.086	0.283
	Reach	0.013	0.875
	Endurance	−0.122	0.127
	Total-AQ®	−0.083	0.301
Age	Control	0.103	0.200
	Origin & Ownership	0.108	0.178
	Reach	0.095	0.235
	Endurance	0.091	0.255
	Total-AQ®	0.140	0.079
Marital status	Control	−0.218^**^	**0.006**
	Origin & Ownership	−0.267^**^	**<0.001**
	Reach	−0.019	0.815
	Endurance	0.009	0.908
	Total-AQ®	−0.156	0.051
Level of education	Control	0.018	0.819
	Origin & Ownership	−0.027	0.737
	Reach	−0.114	0.155
	Endurance	−0.011	0.895
	Total-AQ®	−0.078	0.330
Academic rank	Control	−0.062	0.443
	Origin & Ownership	0.064	0.424
	Reach	0.054	0.503
	Endurance	0.114	0.156
	Total-AQ®	0.065	0.420
College/institute	Control	−0.095	0.236
	Origin & Ownership	−0.075	0.349
	Reach	0.052	0.516
	Endurance	0.047	0.562
	Total-AQ®	−0.002	0.981
Length of service	Control	0.103	0.199
	Origin & Ownership	0.134	0.095
	Reach	0.079	0.327
	Endurance	0.140	0.081
	Total-AQ®	0.178^*^	**0.026**
Working side	Control	0.176^*^	**0.028**
	Origin & Ownership	0.109	0.175
	Reach	0.030	0.706
	Endurance	−0.032	0.691
	Total-AQ®	0.090	0.263

Bold values indicate statistically significant correlations (*P* < 0.05). **Correlation is significant at the 0.01 level (2-tailed).

Internal correlations among the CORE dimensions (Table 3) revealed strong interrelationships. Control was significantly associated with Origin & Ownership (*r* = 0.484, *P* < 0.001), while Reach correlated positively with Endurance (*r* = 0.289, *P* < 0.001). This indicates that faculty who perceive greater control also tend to take ownership of adversity, and those who limit its spread are more likely to view it as temporary.

**Table 3:** Correlation Between AQ® four dimensions (CORE) (*n* = 157).

AQ®-dimensions	Variables	Spearman’s rho coefficient correlation	*P*-Value
Control	Control	1.000	
	Origin & Ownership	0.484^**^	**<0.001**
	Reach	0.118	0.140
	Endurance	0.053	0.506
Origin & Ownership	Control	0.484^**^	**<0.001**
	Origin & Ownership	1.000	
	Reach	0.104	0.195
	Endurance	0.056	0.490
Reach	Control	0.118	0.140
	Origin & Ownership	0.104	0.195
	Reach	1.000	
	Endurance	0.289^**^	**<0.001**
Endurance	Control	0.053	0.506
	Origin & Ownership	0.056	0.490
	Reach	0.289^**^	**<0.001**
	Endurance	1.000	

Bold values indicate statistically significant correlations (*P* < 0.05).

**Correlation is significant at the 0.01 level (2-tailed).

### Level of Adversity Quotient® and its four dimensions with mean and standard deviation

Table 3 presents the correlations between the CORE dimensions of Adversity Quotient® (AQ®).

## Discussion

In summary, the faculty at SMBBMU demonstrated average overall resilience, with particular strengths in Reach and Endurance. Another key finding of this study was that age had no significant relationship with overall AQ® or its CORE dimensions. A study conducted on Egyptian business leaders found no significant correlation between age and AQ®, suggesting that individuals across age groups share a comparable ability to handle challenges[10].

This study comprised 68.2% males and 31.8% females, with no significant relationship found between gender and overall AQ® or its CORE dimensions. Similarly, a study of 400 high school students in Haryana reported no significant link between AQ® and gender[15]. This study found a significant but weak negative correlation between marital status and certain AQ® dimensions. AQ®-Control (*r* = −0.218, *P* = 0.006) and AQ®-Origin & Ownership (*r* = −0.267, *P* < 0.001) were lower among unmarried respondents, though marital status was not a strong predictor. In contrast, a study on married women in semi-urban and urban India found no difference in AQ® between married and unmarried women[9]. The discrepancies between the two studies may stem from geographical variations in the study populations. Furthermore, the current study included both male and female participants, whereas the reference study focused solely on women, which may also explain the differing results.

This study found no significant correlation between education level or academic rank and AQ® scores, including the CORE dimensions. Similarly, a study on prospective higher education students in Gandhinagar, Gujarat, reported no significant differences in AQ® scores based on gender, educational stream, or family-related variables such as family type, size, education level, parental employment, and occupation[16].

This study reported a weak positive correlation between the length of service and overall AQ®, indicating a slight increase in AQ® scores with longer tenure. Similarly, a study at West Visayas State University, Lambunao Campus, found that faculty with over 20 years of service had a “high” AQ®, while those with less than 20 years showed an “average” AQ®[17].

Moreover, we observed a statistically significant positive correlation between the working side and the Control dimension (*r* = 0.176, *P* = 0.028), indicating that faculty members working on the clinical side reported slightly higher perceived control compared to those working on the non-clinical side. However, no significant correlation was found between the work setting and the other CORE dimensions or the overall AQ® score.

Adversity Quotient® (AQ®) reflects the degree of responsibility individuals assume when facing challenges, with higher scores denoting accountability and a proactive mindset[11]. In this study, 40.8% of faculty demonstrated average AQ® levels, while the majority of the remainder fell into below-average or low categories, and only a small fraction achieved above-average or high scores. Within the Control dimension, most respondents clustered in the average range, but nearly 56% scored below average or low, indicating limited perceived influence over adversity. These findings suggest that, although resilience is moderate overall, a substantial proportion of faculty struggle with control and responsibility, highlighting critical areas for institutional support.

AQ®-Control represents the extent to which individuals feel capable of influencing adverse situations; higher scores suggest a proactive orientation and a tendency to reframe challenges as opportunities[18]. In contrast, performance in the Origin & Ownership dimension was notably weaker. A substantial proportion of respondents scored at the lower end, with 42.7% classified as low and 40.1% as below average. Only 15.3% achieved average scores, while above-average (1.3%) and high (0.6%) scores were rare. These findings highlight limited responsibility-taking and reduced perceived influence over adversity among faculty, underscoring this dimension as a critical area for targeted support and intervention.

The Reach dimension reflects the extent to which individuals allow adversity to influence different areas of their lives, with higher scores indicating the ability to contain its impact and maintain control[19]. In this study, most faculty demonstrated moderate to strong performance in Reach, with a substantial proportion achieving average or high scores and relatively few falling into the lower categories. These findings suggest that many faculty members are able to compartmentalize challenges and prevent them from spreading across personal and professional domains, a strength that contributes positively to overall resilience.

The Endurance dimension reflects how individuals perceive the duration of adversity, with higher scores indicating optimism and the belief that challenges are temporary[20]. In this study, nearly half of the faculty scored in the average range, while a notable proportion achieved high or above-average scores, suggesting a generally positive outlook toward adversity. Only a small minority fell into the lower categories, indicating that most respondents were able to view difficulties as manageable and short-lived. This tendency to frame adversity as temporary is an important strength that supports resilience and reduces the risk of prolonged stress. Comparable patterns were observed in the study “Correlation Between Adversity Quotient and Job Performance of LGU Employees of Tayabas City,” where a substantial proportion of respondents scored below average in the Control and Origin & Ownership dimensions, highlighting difficulties in managing adversity and a tendency toward stress and helplessness. In contrast, scores for Reach and Endurance were predominantly average, reflecting moderate resilience but also suggesting a vulnerability to overreaction and diminished optimism when faced with prolonged challenges[21]. These parallels reinforce the current study’s findings, underscoring that weaknesses in control and ownership are common across professional groups and may represent critical targets for resilience-building interventions.

Previous studies indicate that resilience, emotional intelligence, and coping strategies tend to improve with teaching experience and professional maturity. Similar studies from medical schools in Asia and the Middle East have reported that faculty with stronger perceived control and ownership demonstrate lower burnout risk and greater job satisfaction. Research on faculty emotional intelligence also suggests that educators who perceive adversity as manageable and temporary, mirroring the AQ dimensions of Control and Endurance, display reduced emotional exhaustion and better classroom performance[22]. From a practical standpoint, institutions could utilize AQ assessments to design targeted faculty development programs focusing on stress management, mentorship, and constructive coping. Workshops on reflective practice, workload balance, and crisis management could potentially enhance AQ scores, thereby strengthening institutional resilience.

## Limitations

This study had a few limitations that should be acknowledged. The study utilized stratified probability sampling for faculty; however, the colleges themselves were chosen via convenience sampling. This non-random approach at the institutional level may restrict the generalizability of the findings. Second, the cross-sectional design limits causal inference, as AQ® scores were measured at a single time point. Third, the reliance on self-reported data introduces the possibility of social desirability bias, despite measures taken to ensure anonymity.

## Recommendations

Based on the key findings of this study, i.e., consistently low scores in Origin & Ownership, a critical area for targeted institutional support and intervention is highlighted. Counseling services and targeted interventions focusing on the dimensions of Control, Origin & Ownership, Reach, and Endurance are recommended to support personal and emotional growth. Institutions should foster a resilient work culture by reducing administrative burdens, ensuring transparent promotion criteria, and implementing faculty recognition programs. Finally, experimental trials of AQ® training workshops, resilience coaching, and emotional intelligence development modules are suggested for faculty development.

Based on the limitations of this study, we suggest that future research should employ longitudinal designs, broader sampling strategies, and mixed methods, i.e., experimental and longitudinal designs, to strengthen evidence on enhancing AQ® among faculty.

## Conclusion

This study highlights that medical faculty at SMBBMU demonstrate average resilience overall, with strengths in Reach and Endurance but persistent weaknesses in Control and Origin & Ownership, highlighting these as primary areas for improvement. Strengthening the dimensions of Control and Origin & Ownership is essential to enhancing both individual resilience and the overall Adversity Quotient®. Demographic factors showed minimal influence, though marital status and length of service had weak associations. The strong internal correlations between CORE dimensions emphasize their interconnected role in shaping adaptive responses. Targeted interventions, such as resilience workshops, mentorship, and stress management programs, are essential to strengthen responsibility-taking and perceived control. Enhancing these dimensions can improve faculty well-being, institutional resilience, and ultimately the quality of medical education.

## Data Availability

Available upon reasonable request.
